# Effect of human cytomegalovirus (HCMV) US27 on CXCR4 receptor internalization measured by fluorogen-activating protein (FAP) biosensors

**DOI:** 10.1371/journal.pone.0172042

**Published:** 2017-02-16

**Authors:** Jordan M. Boeck, Juliet V. Spencer

**Affiliations:** Department of Biology, University of San Francisco, San Francisco, California, United States of America; University of St Andrews, UNITED KINGDOM

## Abstract

Human cytomegalovirus (HCMV) is a widespread pathogen and a member of the *Herpesviridae* family. HCMV has a large genome that encodes many genes that are non-essential for virus replication but instead play roles in manipulation of the host immune environment. One of these is the US27 gene, which encodes a protein with homology to the chemokine receptor family of G protein-coupled receptors (GPCRs). The US27 protein has no known chemokine ligands but can modulate the signaling activity of host receptor CXCR4. We investigated the mechanism for enhanced CXCR4 signaling in the presence of US27 using a novel biosensor system comprised of fluorogen activating proteins (FAPs). FAP-tagged CXCR4 and US27 were used to explore receptor internalization and recovery dynamics, and the results demonstrate that significantly more CXCR4 internalization was observed in the presence of US27 compared to CXCR4 alone upon stimulation with CXCL12. While ligand-induced endocytosis rates were higher, steady state internalization of CXCR4 was not affected by US27. Additionally, US27 underwent rapid endocytosis at a rate that was independent of either CXCR4 expression or CXCL12 stimulation. These results demonstrate that one mechanism by which US27 can enhance CXCR4 signaling is to alter receptor internalization dynamics, which could ultimately have the effect of promoting virus dissemination by increasing trafficking of HCMV-infected cells to tissues where CXCL12 is highly expressed.

## Introduction

Human cytomegalovirus (HCMV) is a ubiquitous pathogen, with a majority of the human population being seropositive [1]. While HCMV infection is generally asymptomatic, life-threatening disease can occur in immunocompromised individuals, such as organ graft recipients and AIDS patients [2, 3]. Congenital HCMV infection affects 1 in 150 children in the United States and causes neurodegenerative disorders such as hearing loss and blindness [4, 5]. Development of an HCMV vaccine is considered a high priority for improving human health in the United States [6], but these efforts have not yet been successful and a better understanding of how HCMV manipulates the host immune system is necessary to move the vaccine effort forward.

HCMV is a member of the β-subgroup of the *Herpesviridae* family, and these viruses are characterized by a relatively slow replication cycle. Like other herpesviruses, HCMV is capable of remaining in the host and causing life-long latent infection [7]. Despite strict species specificity, HCMV has a broad host cell tropism, infecting epithelial cells of gland and mucosal tissue, smooth muscle cells, fibroblasts, macrophages, dendritic cells, hepatocytes and vascular endothelial cells, and therefore, the virus has multiple routes for systemic spread within the host and between hosts [8]. The HCMV genome is one of the largest among human viruses with over 236 kb of linear DNA and 192 annotated open reading frames (ORFs) [9]. Only a subset of these genes are essential for virus replication [10], while others function to modify host cellular activities in ways that promote virus dissemination, persistence, and evasion of immune clearance. In particular, HCMV encodes four G protein-coupled receptors (GPCRs), US27, US28, UL33, and UL78, which share homology to human chemokine receptors [11, 12].

US28 is regarded as a true chemokine receptor that binds and signals in response to multiple host chemokines including CX_3_CL1/Fractalkine, CCL2/MCP-1, CCL5/RANTES, and CCL7/MCP-3 [13–16]. In contrast, US27, UL33, and UL78 are currently considered orphan receptors, having no affinity or known response to chemokine treatment [17, 18]. US27, US28, and UL33 have all been found in HCMV virions, dense bodies, and non-infectious extracellular particles (NIEPs) [19–23], suggesting that upon virus fusion with the cell membrane, these viral GPCRs could immediately influence cell signaling networks.

While UL33 and UL78 homologs exist in the genomes of rodent CMVs and have been shown to play roles in virus dissemination [24–27] and latency [28, 29], US27 and US28 are found only in primate CMVs. Consequently, these gene products have been studied mainly *in vitro*, and generally in transfected cells. US27, US28, and UL33 have been found to be predominantly localized to late endosomes, and antibody feeding experiments indicated that both US27 and US28 are constitutively and rapidly endocytosed [19, 20, 30]. A virus mutant lacking US27 limited the virus to direct cell-to-cell spread, suggesting that US27 is required for spreading via the extracellular route [31], which is consistent with the US27 protein being present in the virus particle.

Many GPCRs are capable of physically associating as homodimers or in heteromeric complexes, which can have effects on their signaling and trafficking properties, and HCMV GPCR have been reported to form heteromeric complexes [32, 33]. US28, which can also signal constitutively and activates NF-κB signaling, was found to form heterodimers with each of the other HCMV GPCR [33]. Heterodimerization of US28 with either UL33 or UL78 impaired activation of NF-κB, whereas complex formation with US27 had no effect on NF-κB signaling [33]. HCMV GPCR also interact with cellular receptors, and UL33 or UL78 were found to form oligomers with CXCR4 that significantly reduced signaling and migration responses to ligand CXCL12 (stromal derived factor-1 alpha, or SDF-1α), as well as inhibit the ability of CXCR4 to function as a co-factor for HIV entry [32]. In direct contrast, US27 was found to enhance the expression and signaling activity of CXCR4 [18]. Specifically, US27 increased CXCR4 protein levels, which led to an increase in calcium flux and chemotaxis in response to CXCLl2 [18]. This up-regulation was not observed when CXCR4 was co-expressed with US28 or with US27 mutants lacking either the C-terminal intracellular tail or the DRY box motif in the 2^nd^ intracellular loop, suggesting that these regions of US27 are necessary to facilitate enhanced CXCR4 signaling.

CXCR4 is a chemokine receptor that has been extensively studied as a co-receptor for HIV entry as well as for a role in promoting metastasis in several cancers [34–36]. Upon CXCL12 binding to CXCR4, several G protein-dependent signaling pathways are activated, including calcium mobilization, transcription, and chemotaxis [37]. CXCR4 is essential for vascular development, and mice lacking CXCR4 die *in utero* [38]. Autosomal dominant mutations in CXCR4 that prevent receptor inactivation can lead to *W*art, *H*ypogammaglobulinemia, *I*nfection, and *M*yelokathexis, or WHIM syndrome [39–41]. WHIM patients are immunodeficient due to a chronic reduction of circulating leukocytes, as enhanced CXCL12-induced chemotaxis mediated through CXCR4 results in hematopoietic cells being retained in the bone marrow. Myelokathexis refers to the unusual pathology of the bone marrow, which becomes packed with neutrophils that are unable to enter the bloodstream due to high affinity binding between CXCR4 and CXCL12. Individuals with this condition are highly susceptible to bacterial and viral infections, particularly with strains of human papillomavirus that cause warts on the hands and feet [42].

**Fig 1:**
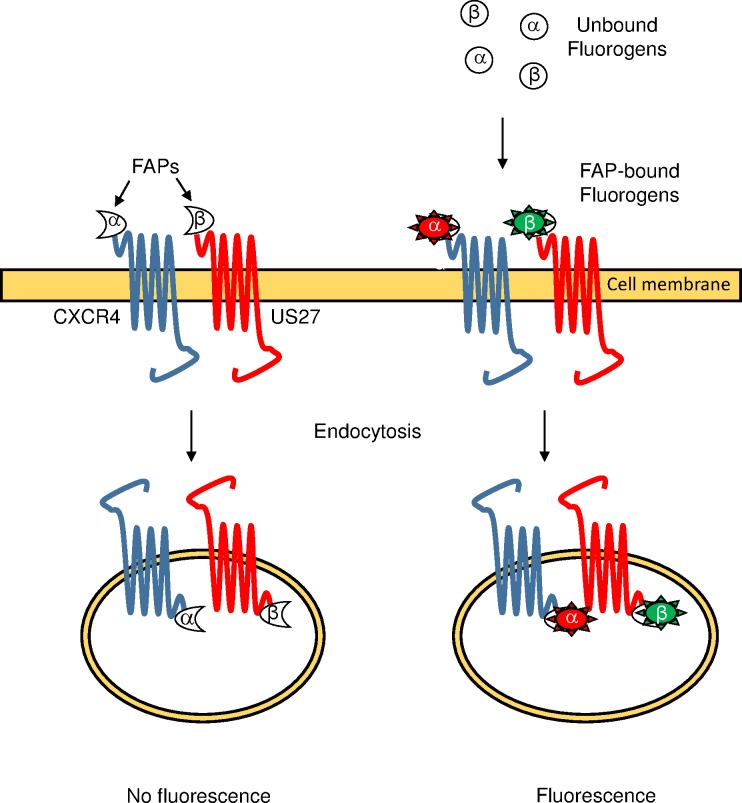
The fluorogen-activating protein (FAP) biosensor system. CXCR4 is expressed as a fusion protein with α-FAP and US27 is expressed as a fusion protein with β-FAP, which each bind with high affinity to distinct membrane impermeable fluorogen dyes. When extracellular fluorogen is not bound to the FAP, neither component exhibits fluorescence, and intracellularly localized FAP-tagged receptors are not exposed to membrane impermeable fluorogens. Upon addition of fluorogens to cell expressing FAP-tagged receptors, binding occurs rapidly and fluorescence can be measured using flow cytometry or fluorescence microscopy. Endocytosed receptors retain the bound fluorogen labels they acquired while present on the cell surface, allowing tracking of receptors from the surface to the cell’s interior.

To investigate the mechanism for enhanced CXCL12-induced CXCR4 signaling by HCMV US27, we hypothesized that US27 might affect the dynamics of CXCR4 receptor internalization. For example, if CXCR4 endocytosis was reduced through sequestering of intracellular machinery by US27, this could prevent CXCR4 desensitization and result in increased signal amplitude or more prolonged signaling outcomes, as in WHIM syndrome. We employed a recently developed biosensor system known as fluorogen activating proteins (FAPs) [43, 44] to study CXCR4 and US27 receptor trafficking. This system consists of two components, a fluorogen and a fluorogen activating polypeptide derived from a single chain antibody fragment (FAP) that is fused to the protein of interest (Fig 1). When the two components are separate, fluorescence is not observed. But upon addition of the fluorogen, rapid binding to the FAP occurs and fluorescence can immediately be measured with flow cytometry or confocal microscopy to monitor receptor trafficking [45–47]. One significant advantage of the FAP system over GFP labeling is that the use of membrane impermeable fluorogen dyes enables labeling of receptors at the cell surface only and tracking of endocytosis from the cell surface. Here, we used FAP-tagged receptors to demonstrate that co-expression of US27 increases ligand-induced but not steady state internalization of CXCR4, resulting in amplified downstream cellular responses to CXCL12.

## Materials and methods

### Cells and reagents

NIH-3T3 mouse fibroblasts stably expressing α-FAP-tagged human CXCR4, β-FAP-tagged HCMV US27 (Genbank Accession ABG73087) or both were purchased from Spectra Genetics (Pittsburgh, PA) and prepared as described previously [48]. Cells were maintained in Dulbecco’s Modified Essential Medium (DMEM) supplemented with 10% fetal bovine serum (FBS; Atlanta Biologicals, Lawrenceville, GA) at 37°C in a humidified 5% CO_2_ atmosphere chamber. Membrane impermeable αRED, βRED, and βGREEN fluorogens were purchased from Spectra Genetics, and purified recombinant human CXCL12 was from Peprotech (Rocky Hill, NJ).

### Flow cytometry

NIH-3T3 cells expressing FAP-tagged receptors were seeded in six-well plates at a density of 2 x 10^5^ cells per well prior to treatment with phosphate buffered saline (PBS) or CXCL12 at the indicated concentration and time points at 37°C. Cells were washed twice with FACS buffer (PBS + 1% BSA and 1% sodium azide), harvested with Cellstripper (Corning, Tewksbury MA), washed twice with FACS buffer, and then labeled with 100nM membrane impermeable αRED or βGREEN fluorogens for 15 minutes on ice. Cells were analyzed using the BD Accuri C6 flow cytometer (BD Biosciences, San Jose, CA).

### Calcium mobilization

Cells were harvested and resuspended in 5mL calcium loading buffer (RPMI + 25mM HEPES) at a density of 2 x 10^6^ cells per ml. Cells were labeled with 1μM Fluo-4AM (Invitrogen, Carlsbad, CA) for 30 minutes at 37°C in the dark with gentle agitation every 10 minutes. Following incubation, samples were washed with calcium loading buffer, centrifuged for 5 minutes at 1000 rpm, and then resuspended in 600 μl calcium loading buffer. Cells were analyzed by flow cytometry, with cells collected for 20 seconds to establish a baseline, and then samples were treated with 100 ng/ml CXCL12 and cells collected for an additional 80 seconds. The calcium ionophore ionomycin (Adipogen, San Diego, CA) was used at 1 mg/ml as a positive control for Fluo-4 loading, and PBS served as a vehicle control. Data was analyzed using FlowJo software (FlowJo, Ashland, Oregon).

### Confocal microscopy

Cells were seeded in four chamber, glass-bottom dishes (Greiner Bio-One, Monroe, NC) at a density of 5 x 10^4^ cells per compartment and then incubated for 24 hours at 37°C. Cells were labeled with 100 nM membrane impermeable fluorogen for five minutes before imaging, followed by treatment with PBS or 100 ng/ml CXCL12 at room temperature. Z-stack images were taken with a Zeiss LSM700 laser scanning confocal fluorescence microscope equipped with an AxioCAM imaging system and Zen software (Carl Zeiss, Inc., Oberkochen, Germany) at 63x objective magnification with immersion oil. Intracellular fluorescence intensity was measured using Image J software.

## Results and discussion

### Expression of US27 and CXCR4-FAP fusion proteins

**Fig 2:**
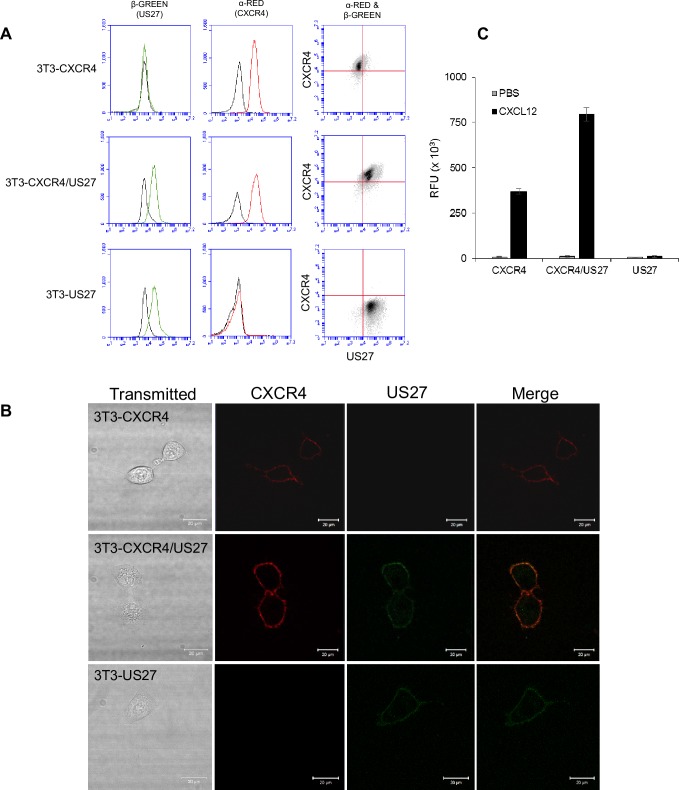
NIH-3T3 cells express FAP-tagged CXCR4 and US27 receptors. (A) NIH-3T3 mouse fibroblasts were stably transfected with plasmids encoding αFAP-tagged CXCR4 and βFAP-tagged US27. Cells were labeled with membrane impermeable α and β fluorogens, and surface expression of each receptor measured via flow cytometry. The red histogram represents CXCR4, blue histogram is US27, and black histogram is unstained cells. (B) Cells were seeded in a glass-bottom dish, labeled with membrane impermeable α and β fluorogens, and imaged immediately using a Zeiss LSM700 laser scanning confocal microscope. Scale bar, 20 μm. (C) Cells were labeled with Fluo-4 AM calcium indicator dye, then stimulated with either PBS or 100 ng/ml CXCL12/SDF and fluorescence intensity was measured with flow cytometry. RFU = relative fluorescence units. Error bars represent standard error of three replicate experiments.

In order to examine effects of US27 on endocytosis of CXCR4, we first analyzed expression of αFAP-tagged human CXCR4 in NIH-3T3 cells in the presence or absence of βFAP-tagged HCMV US27. Treatment with αRED dye labeled αFAP-CXCR4 receptors on the cell surface, as indicated by flow cytometry (Fig 2A).

US27 surface levels were visualized with the addition of βGREEN dye (Fig 2A). In addition, cells were cultivated in glass bottom dishes and live cell imaging commenced immediately following dye addition (Fig 2B). αFAP-tagged CXCR4 was evident on the surface of both 3T3-CXCR4 and 3T3-CXCR4/US27 cells (red), and βFAP-tagged US27 was present in 3T3-US27 cells and 3T3-CXCR4/US27 cells (green). The level of US27 expression was comparable between 3T3-CXCR4/US27 and cells expressing US27 only, while the level of CXCR4 expression appeared slightly higher in the CXCR4/US27 cells compared to CXCR4 only cells (Fig 2B). These results demonstrate expression of FAP-tagged receptors on the epithelial cell surface.

### US27 enhances CXCL12-induced calcium mobilization of FAP-tagged CXCR4

To confirm that αFAP-CXCR4 was functional and responsive to human CXCL12 treatment, calcium flux was measured by flow cytometry using Fluo-4 calcium sensitive dye. CXCL12 binding to CXCR4 induced a rapid and transient release of calcium ions, as evidenced by the rapid increase in fluorescence intensity (Fig 2C). We previously observed that in HEK293 cells, the level of calcium mobilization induced by CXCL12 was greater when CXCR4 was co-expressed with US27, but not US28 [18]. Similarly, we found here that 3T3-CXCR4/US27 cells exhibited higher peak fluorescence intensity upon stimulation with CXCL12 than 3T3-CXCR4 cells, corresponding to greater calcium flux in these cells (Fig 2C). There was no increase in fluorescence upon addition of CXCL12 to 3T3-US27 cells, confirming that US27 alone does not induce calcium flux in response to this chemokine. PBS served as a vehicle control and yielded no increase in fluorescence (Fig 2C), while ionomycin was used as a positive control for labeling (not shown). These results confirm that the potentiating effect of US27 on CXCR4 signaling occurs in multiple cell types, and also demonstrate that the FAP-tagged receptors exhibit signaling patterns comparable to the native receptors.

### US27 increases CXCL12-induced CXCR4 internalization

**Fig 3:**
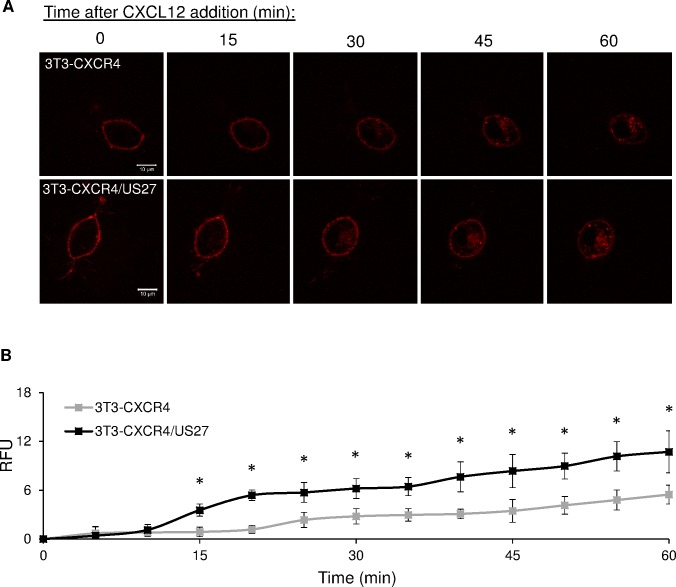
US27 increases CXCL12-induced endocytosis of CXCR4. (A) Confocal images of 3T3-CXCR4 and 3T3-CXCR4/US27 cells grown in glass-bottom dishes and labeled with 100 nM membrane impermeable αRED fluorogen after treatment with 100 ng/ml CXCL12. Scale bar, 10 μm. (B) Total intracellular fluorescence of cells quantified from images taken every 5 minutes (a subset of which is shown in A) with Image J software. Error bars are standard error from average measurement of five cells. * indicates p<0.05, Student t test.

To investigate the impact of US27 on the rate of endocytosis by CXCR4, we used live cell imaging to observe receptor internalization. FAP-tagged NIH/3T3 cells were seeded in glass-bottom dishes, labeled with membrane impermeable αRED fluorogen, and then stimulated with CXCL12. CXCR4 internalization was evident by the red punctae that accumulated in the cell’s interior (Fig 3A).

These punctae likely represent receptors that had been on the surface, bound αRED dye, and then were endocytosed in response to ligand CXCL12. The amount of fluorescence that accumulated inside 3T3-CXCR4/US27 cells was greater than for 3T3-CXCR4 cells at each time interval (Fig 3A), suggesting that US27 enhanced internalization of CXCR4. As expected, the brightness of the fluorescent outline of the cell membrane decreased over time as more fluorogen-bound receptors were internalized for both cell lines. Quantification of intracellular fluorescence indicated that more CXCR4 was internalized upon CXCL12 binding in the presence of US27, compared to cells expressing CXCR4 only (Fig 3B). Treatment with PBS served as a control, and the level of constitutively internalized CXCR4 was found to be comparable in the presence or absence of US27 (S1 Fig). This suggests that US27’s effects on CXCR4 are specific to ligand-induced internalization and signaling and do not impact steady state receptor recycling.

### US27 is rapidly and constitutively internalized independently of CXCR4 or CXCL12

**Fig 4:**
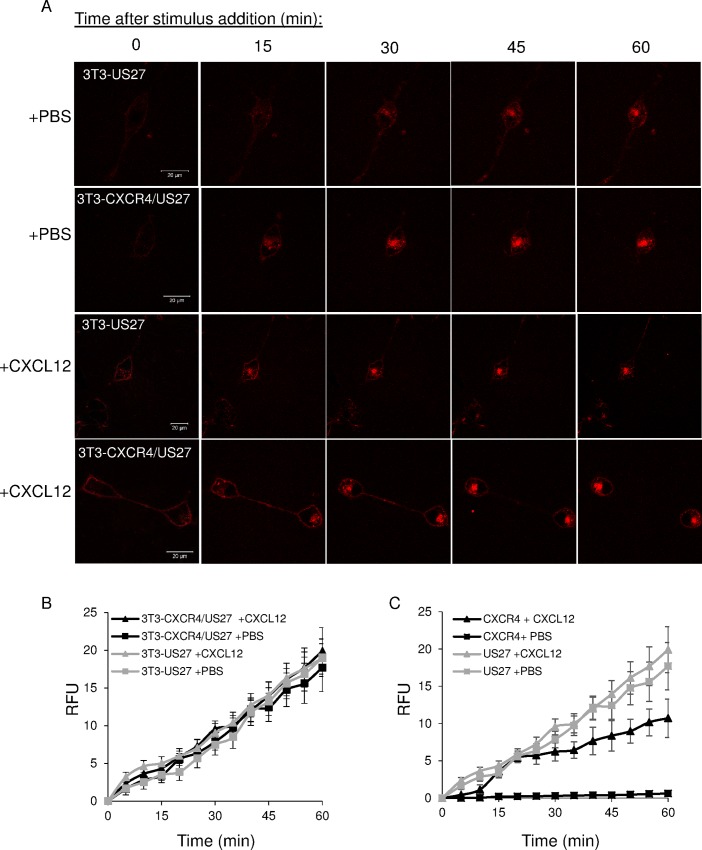
US27 is rapidly and constitutively internalized independent of CXCR4 or CXCL12. (A) Live cell imaging after labeling with 100 nM membrane impermeable βRED and treatment with either PBS or 100 ng/ml CXCL12. Scale bar, 20 μm. (B) Quantification of US27 intracellular fluorescence levels in images acquired every five minutes in 3T3-US27 (gray) or 3T3-CXCR4/US27 cells (black). (C) Comparison of US27 (gray) and CXCR4 (black) internalization rates in 3T3-CXCR4/US27 cells. Error bars are standard error from average measurement of five cells.

Since US27 altered CXCR4 internalization dynamics, we next investigated whether CXCR4 impacted US27 internalization. 3T3-US27 and 3T3-CXCR4/US27 cells in glass-bottom dishes were stained with membrane impermeable βRED fluorogen, and then treated with CXCL12. Live cell imaging revealed that the amount of US27 internalized was comparable in the presence or absence of CXCR4 (Fig 4A). While CXCR4 internalization increased dramatically upon CXCL12 binding (Fig 3A and 3B), US27 internalization was not affected by the addition of the chemokine (Fig 4A and 4B). These results demonstrate that US27 is rapidly and constitutively internalized, and although US27 increased CXCR4 internalization, there was no reciprocal effect on US27 by CXCR4. The relative rates for both CXCL12-induced and constitutive endocytosis were significantly higher for US27 than for CXCR4 when both receptors were measured in the same cell (Fig 4C).

### CXCR4 and US27 are co-localized at the plasma membrane and inside the cell

**Fig 5:**
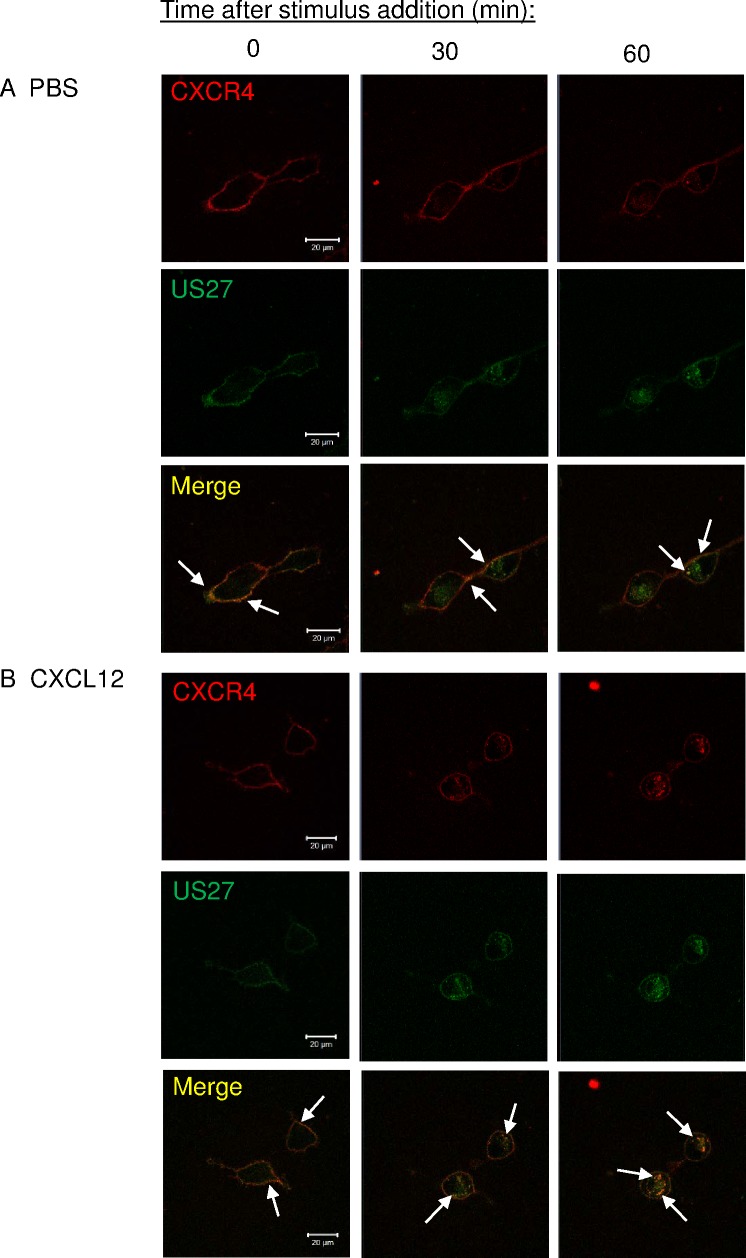
US27 colocalizes with CXCR4 after CXCL12 treatment but also internalizes independently. 3T3-CXCR4/US27 cells were seeded in glass-bottom dishes, labeled with 100nM membrane impermeable αRED (CXCR4) and βGREEN (US27) fluorogens, and then treated with (A) PBS or (B) 100 ng/ml CXCL12. Images were acquired every five minutes with representative images shown. Scale bar, 20 μm. White arrows indicate discrete yellow punctae indicating overlapping red and green signal.

To examine whether CXCR4 and US27 were internalized in the same endocytic vesicles, 3T3-CXCR4/US27 cells were seeded in glass-bottom dishes, stained with both membrane impermeable αRED and βGREEN fluorogens, and treated with either CXCL12 or PBS. Far more US27 was internalized than CXCR4 when the stimulus was PBS (Fig 5A), as evidenced by the accumulation of mainly green fluorescence inside the cell. There was some receptor co-localization evident, visualized as yellow staining where overlapping red and green fluorescence occurred, primarily in the membrane outline at the cell surface, but also in at least one intracellular punctae (Fig 5A, arrows). Stimulation with CXCL12 induced endocytosis of more CXCR4 than PBS, and this resulted in greater intracellular yellow staining in discrete punctae (Fig 5B), suggesting CXCR4 was internalized within the same endocytic vesicles as US27.

### US27 delays CXCR4 surface recovery

**Fig 6:**
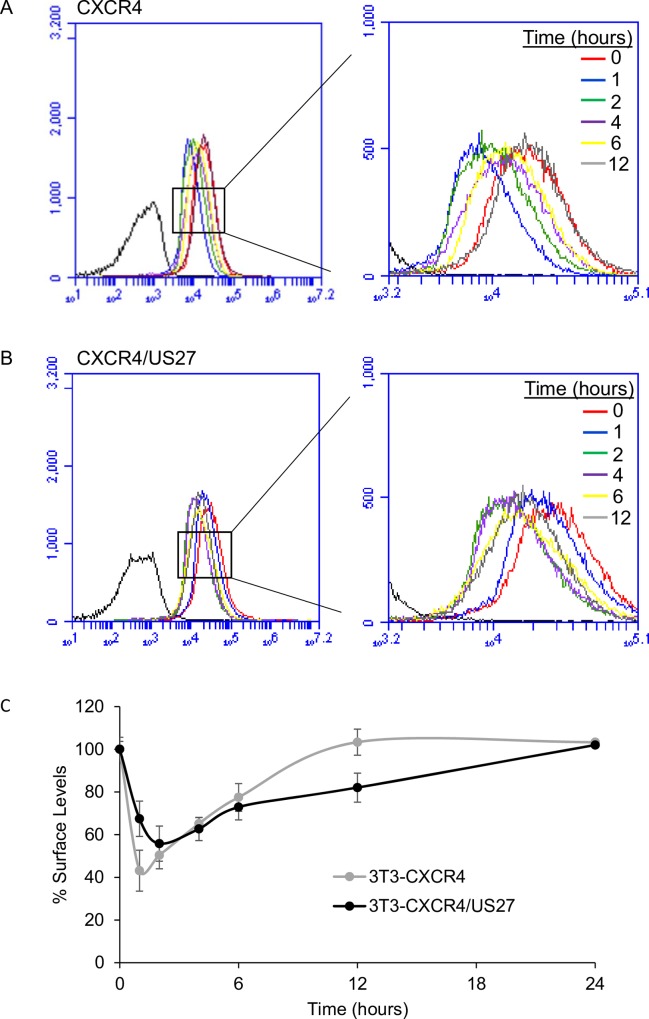
US27 delays CXCR4 surface recovery. (A) 3T3-CXCR4 and (B) 3T3-CXCR4/US27 cells were treated with 100 ng/ml CXCL12 or PBS for the indicated time points, harvested, and then labeled with membrane impermeable αRED fluorogen and fluorescence intensity was measured via flow cytometry. (C) Percent of total initial surface levels for CXCR4 at each time interval. Error bars indicate standard error among three replicate experiments.

The rate of CXCR4 recovery back to the cell surface was evaluated also over time in the presence and absence of US27 by using flow cytometry. Cells were stimulated with CXCL12, stained at various time points post-stimulation, and then surface receptor levels were evaluated. For 3T3-CXCR4 cells, the largest decrease in fluorescence intensity occurred within the first hour post-stimulus (Fig 6A). By the second hour, an increase in fluorescence was observed, suggesting that CXCR4 was recycling back to the cell surface, which gradually continued until 12 hours post-stimulus. After 12 hours, CXCR4 levels were completely restored to initial levels. In contrast, when US27 was present, significant CXCR4 internalization occurred within the first hour but maximal internalization was observed at two hours post-stimulation (Fig 6B). Slight cell surface recovery of CXCR4 was first noted at four hours and gradually continued, yet recovery was still not complete at 12 hours. Evaluation of longer time periods showed that complete recovery took 24 hours (Fig 6C), indicating that US27 may slow CXCR4 return to the cell surface. Although a single concentration of 0.1 μg/ml CXCL12 was employed in these experiments, similar results were observed with doses ranging from 0.001–0.5 μg/ml (S2 Fig).

### US27 surface levels remain relatively constant over time

**Fig 7:**
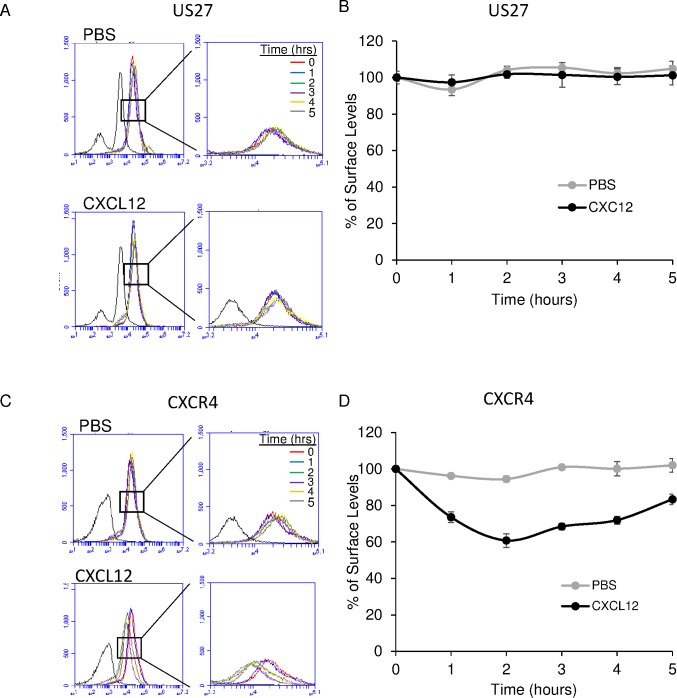
US27 surface levels remain constant over time. 3T3-CXCR4/US27 cells were treated with 100 ng/ml CXCL12 or PBS for the indicated times, harvested, and then labeled with membrane impermeable fluorogen and analyzed via flow cytometry. (A) αRED fluorescence labeling of surface US27. (B) Fluorescence intensity pre-treatment was set as 100% and intensity at each time point expressed as % initial level. (C) αRED labeling of surface CXCR4 and (D) percent total initial surface levels calculated as above. Error bars represent standard error among three independent replicate experiments.

Because US27 was clearly undergoing constitutive internalization, we wondered how much cell surface levels of US27 changed over time. To explore this, 3T3-CXCR4/US27 cells were treated with CXC12 or PBS and labeled with α and β fluorogens at various times post-treatment. No significant changes in fluorescence were observed, as indicated by the overlapping histograms for each treatment (Fig 7A), suggesting that the receptors being endocytosed were replaced by new receptors at a constant rate. For all time points tested, the percentage of total surface US27 fluctuated only minimally from initial levels (Fig 7B), and even the addition of CXCL12, which induced rapid endocytosis of CXCR4 (Fig 7C and 7D), did not affect US27 internalization. These results demonstrate that US27 surface levels remain relatively constant over time, regardless of the stimulant added or other receptors being endocytosed.

## Conclusions

The most successful pathogens have very intricate relationships with their hosts. HCMV has the ability to persist in its host as a lifelong latent infection in part due to extensive manipulation of the host chemokine system [49]. We have previously reported that US27 modulates the signaling of host chemokine receptor CXCR4 [18], and in this study we expanded these observations by examining the impact of US27 on CXCR4 endocytosis and recycling dynamics. Using the FAP biosensor system, we found that US27 increases CXCR4 receptor internalization following ligand binding and slows recycling back to the surface, ultimately increasing calcium mobilization and the downstream signaling output resulting from CXCL12 engagement of CXCR4.

The FAP system is a valuable tool for fluorescently labeling proteins of interest with minimal unspecific labeling. While GFP-tagging is a well-established method for tracking the movement of specific proteins, one disadvantage of GFP-proteins is that they exhibit fluorescence regardless of their cellular location, making it challenging to distinguish surface-localized protein from intracellular protein. Even highly sensitive light microscopy techniques such as total internal reflection fluorescence microscopy (TIRF) cannot distinguish proteins that are in the plasma membrane from those that are in vesicles within 50 nm of the surface [50, 51]. In the FAP system, fluorescent signal depends on the interaction between a reporter polypeptide and a small membrane impermeable molecule that fluoresces only when bound to the reporter. This system has been successfully used to study GPCR internalization, trafficking, and drug screening using flow cytometry and fluorescence microscopy [43, 45, 52]. To our knowledge, this is the first study using FAPs to observe real-time protein trafficking of a virally encoded GPCR. Moreover, FAP internalization could provide a useful readout for ligand screening and “de-orphanizing” viral GPCR with unknown signaling outcomes like US27, UL33, and UL78, as well as other orphan cellular receptors. Although we took advantage of existing murine fibroblasts that expressed αFAP-tagged human CXCR4 [48] for this study, FAP-tagged receptors can also be expressed in a variety of cell types that are more relevant to HCMV infection, such as human epithelial cells and monocytes.

We have examined the effects of US27 on CXCR4 here using transfected proteins, in the absence of virus infection. This characterization is a necessary first step towards understanding the functions of US27 because HCMV infection induces many changes in the host cell and it can be difficult to isolate the effects of individual viral proteins. During infection, potentiation of CXCR4 signaling by US27 might promote increased migration of infected cells towards CXCL12-expressing tissues, such as the bone marrow. As the bone marrow is the primary site of latency [53], trafficking of infected cells to this site could deliver virus that infects newly developed myeloid progenitors, thus expanding the pool of latently infected cells and ensuring long term virus persistence. However, it is also possible that during infection, other viral genes may modify or ablate the effects of US27 on CXCR4 signaling, particularly UL33 and UL78, which have been found to associate with CXCR4 and impair CXCL12-induced signaling outcomes [32]. Indeed, during lytic infection, US27 is expressed with relatively late kinetics [17], when the actin cytoskeleton has been remodeled, and several groups have reported significantly reduced migration by infected cells [54–58]. Conversely, Smith et al. [59] found that infection of primary human monocytes increased trans-endothelial migration and cell movement. Because the US27 protein is also present in the virion [22, 23] it is possible that the viral GPCR may have immediate effects on cell signaling after virus entry and influence CXCR4 at an early stage of infection. Future studies are necessary to examine the impact of US27 on CXCR4 during infection in various cell types to determine whether US27 actually promotes increased migration of infected cells towards CXCL12.

HCMV has the ability to infect a wide range of cell types, and the virus disseminates to various organs in the human body. Control of the host chemokine system appears to be crucial for this process, as the virus encodes not only four chemokine receptor-like GPCR, but also a functional chemokine, UL146, that is chemotactic for neutrophils [60]. UL146 has been postulated to aid in virus dissemination through recruitment of neutrophils to sites of infection. This possibility has yet to be examined in the context of virus infection *in vivo*, mainly because the species specificity of HCMV remains a barrier to its use with animal models. The natural infection of *Rhesus macaques* by Rhesus cytomegalovirus (RhCMV) is an excellent model system well-suited for the examination CMV-host interactions [61], but primate experimentation is extremely expensive, and research in these models has focused on mainly on vaccine efficacy to date. In clinical isolates from infected humans, the UL146 gene is among the most highly variable in the HCMV genome [62, 63], although specific polymorphisms have not yet been linked to disease outcome or severity [62, 64]. Additional work is needed to fully understand the mechanisms by which HCMV controls cell migration patterns to promote virus dissemination and persistence.

On a cellular level, receptor trafficking is an important process and there are many associated proteins that also play major roles in signaling networks. While we have demonstrated that US27 affects the internalization kinetics of CXCR4, we have not yet explored the role of any associated proteins, such as arrestins, G protein receptor kinases (GRKs), or Rab GTPases [37]. Intriguingly, we found that CXCR4 expression levels were moderately higher in NIH3T3 mouse fibroblasts transfected with FAP-tagged US27 and CXCR4 compared to cells expressing only CXCR4 (Fig 2A), which is consistent with previous observations of increased CXCR4 expression in the presence of US27 in transfected HEK293 cells [18]. Thus, in addition to the changes in receptor endocytosis dynamics reported here, there are likely other mechanisms by which US27 impacts CXCR4 activity, such as regulation of transcription, mRNA stabilization, or reduced protein degradation.

In summary, we used the FAP system with flow cytometry and live cell imaging to show that cells co-expressing CXCR4 and HCMV US27 exhibit higher CXCR4 levels, greater CXCL12-induced internalization, and slower CXCR4 receptor recovery back to the cell surface. This work has revealed a novel regulatory function for an orphan viral receptor, US27, which could have implications for the maintenance of HCMV latency.

## Supporting information

S1 Fig US27 does not impact CXCR4 constitutive internalization. 3T3-CXCR4 and 3T3-CXCR4/US27 cells in glass-bottom dishes were labeled with 100nM αRED, and then treated with PBS and images acquired at 5 minute intervals. The above figure represents a subset of these images from one representative experiment that was done in triplicate. Scale bar, 20 μm.
(TIF)

S2 Fig FAP-tagged CXCR4 is responsive to CXCL12. NIH3T3 cells were treated with the indicated doses of CXCL12 for 1 hr, then labeled with membrane impermeable αRED fluorogen. Fluorescence intensity was measured using flow cytometry and expressed as a percentage of initial surface level. Error bars represent standard error among three replicate experiments.
(TIF)
