# Supplementary figures and images for: Effect of human cytomegalovirus (HCMV) US27 on CXCR4 receptor internalization measured by fluorogen-activating protein (FAP) biosensors
Source: PLoS One. 2017 Feb 16;12(2):e0172042. doi: 10.1371/journal.pone.0172042 (PMC5313195; doi:10.1371/journal.pone.0172042)

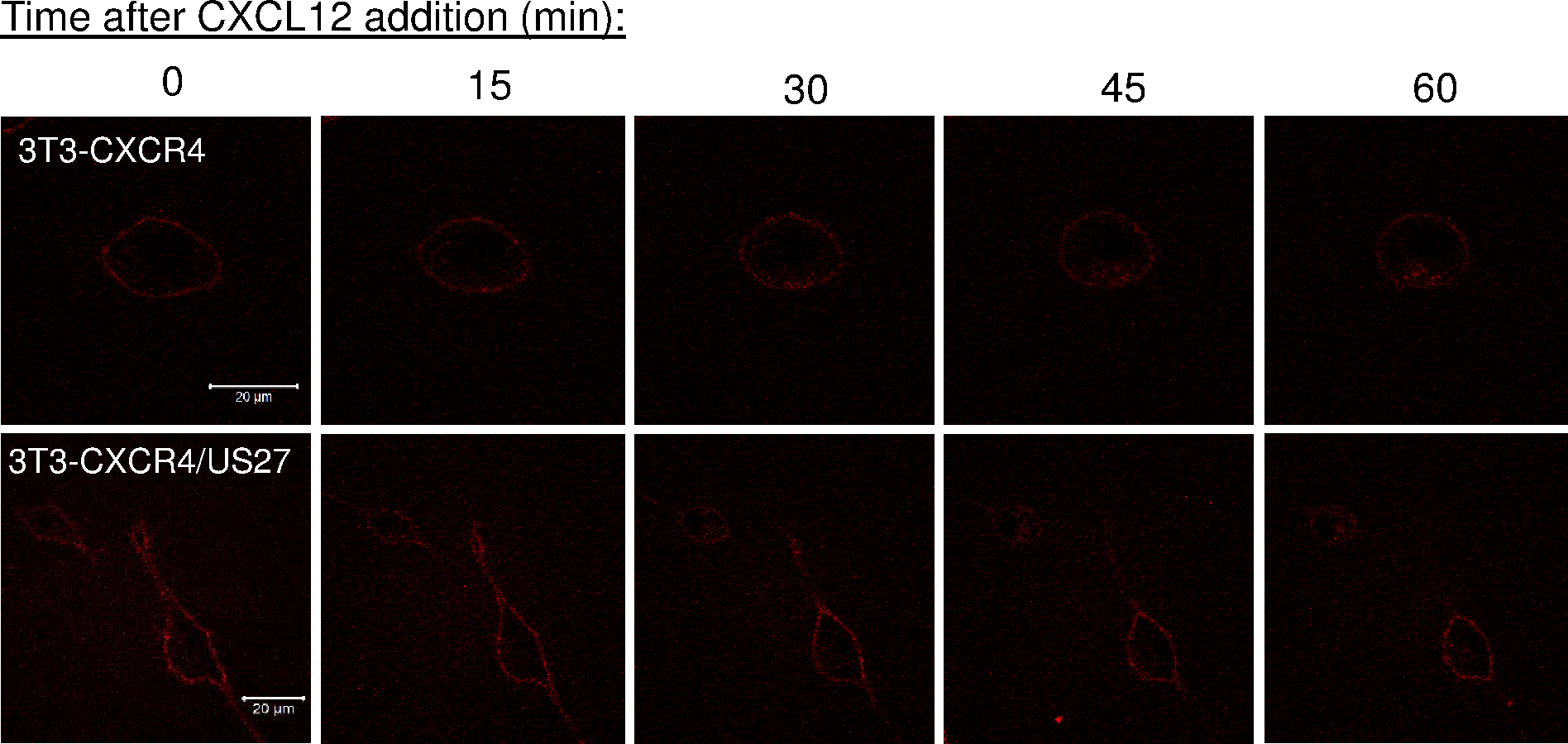

Supplement: S1 Fig — 3T3-CXCR4 and 3T3-CXCR4/US27 cells in glass-bottom dishes were labeled with 100nM αRED, and then treated with PBS and images acquired at 5 minute intervals. The above figure represents a subset of these images from one representative experiment that was done in triplicate. Scale bar, 20 μm. (TIF) [file pone.0172042.s001.tif]

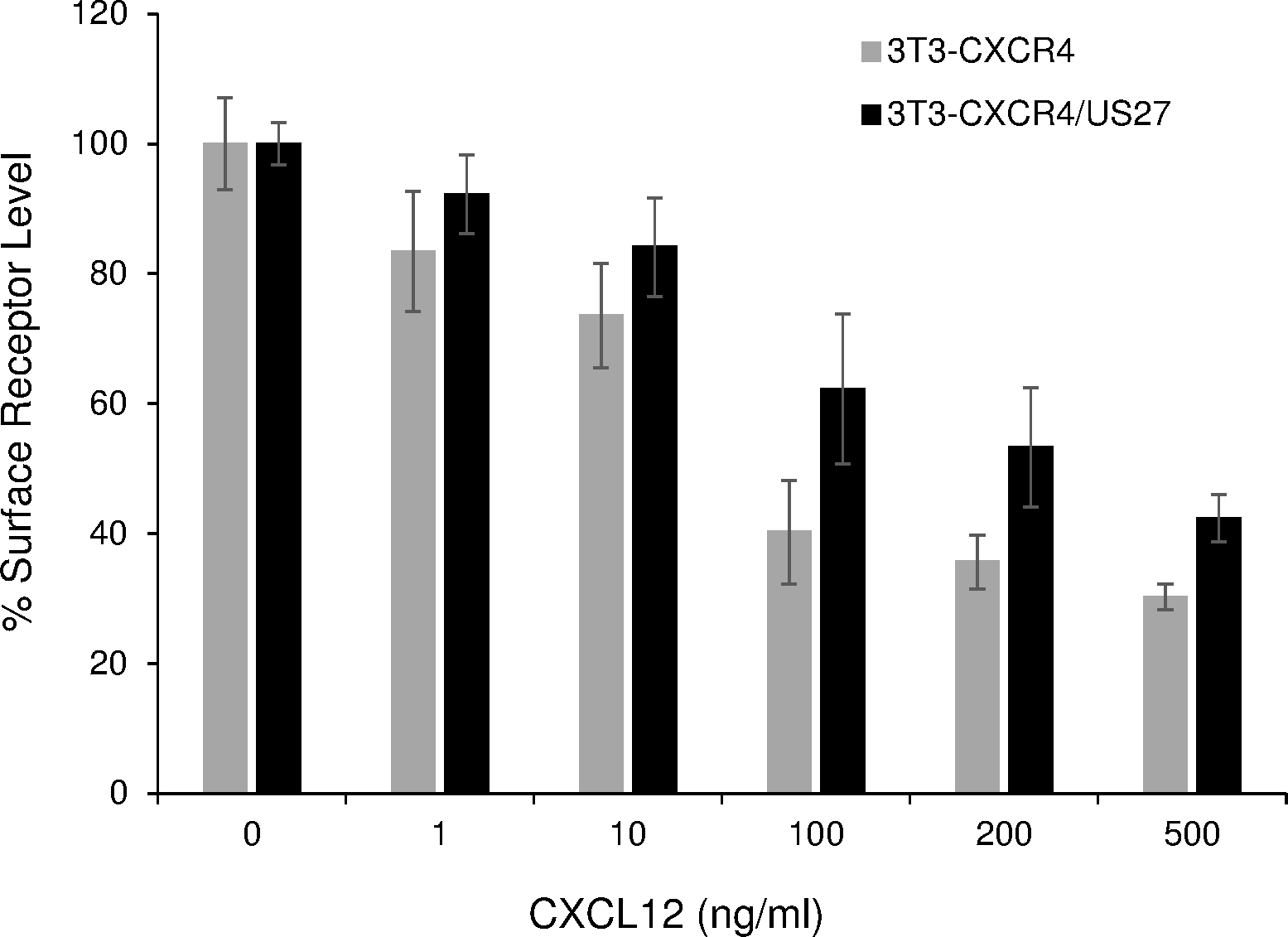

Supplement: S2 Fig — NIH3T3 cells were treated with the indicated doses of CXCL12 for 1 hr, then labeled with membrane impermeable αRED fluorogen. Fluorescence intensity was measured using flow cytometry and expressed as a percentage of initial surface level. Error bars represent standard error among three replicate experiments. (TIF) [file pone.0172042.s002.tif]
